# CD44v9 is associated with epithelial‐mesenchymal transition and poor outcomes in esophageal squamous cell carcinoma

**DOI:** 10.1002/cam4.1874

**Published:** 2018-11-26

**Authors:** Daisuke Taniguchi, Hiroshi Saeki, Yuichiro Nakashima, Kensuke Kudou, Ryota Nakanishi, Nobuhide Kubo, Koji Ando, Eiji Oki, Yoshinao Oda, Yoshihiko Maehara

**Affiliations:** ^1^ Department of Surgery and Science, Graduate School of Medical Sciences Kyushu University Fukuoka Japan; ^2^ Department of Anatomic Pathology, Pathological Science, Graduate School of Medical Sciences Kyushu University Fukuoka Japan

**Keywords:** CD44v9 antigen, epithelial‐mesenchymal transition, esophageal squamous cell carcinoma, neoplastic stem cells, patient outcomes

## Abstract

CD44 serves as a marker of cancer stem cells. Alternative splicing generates the CD44v9 isoform. Cancer stem cells are associated with the epithelial‐mesenchymal transition in cancers, although little is known about their role in esophageal squamous cell carcinoma. Here, we aimed to clarify the relationship between CD44v9 expression, the epithelial‐mesenchymal transition, and clinicopathological features of patients with esophageal squamous cell carcinoma. CD44v9 levels were higher at the tumor invasive front compared with the center of the tumor and higher in metastatic lymph nodes compared with primary tumors. High levels of CD44v9 at the tumor invasive front were significantly associated with deeper tumor invasion and shorter overall survival and recurrence‐free survival. The expression of CD44v9 was increased by treatment with transforming growth factor‐β, which induced esophageal squamous cell carcinoma cells to undergo the epithelial‐mesenchymal transition. Moreover, inhibition of CD44v9 expression decreased the migration and invasiveness of esophageal squamous cell carcinoma cells. These results indicate that the expression of CD44v9 at the tumor invasive front induced by stemness was strongly associated with the epithelial‐mesenchymal transition and poor prognosis of patients with esophageal squamous cell carcinoma. CD44v9 may therefore serve as a novel prognostic biomarker and a potential therapeutic target for esophageal squamous cell carcinoma.

## 1 INTRODUCTION

Esophageal cancer (EC) is the eighth most common cancer worldwide and the sixth most common cause of death from cancer.^1^ Although recent advances in multidisciplinary therapies including chemotherapy, radiotherapy, and surgery have improved the clinical outcomes of patients,^2^, ^3^ EC remains a challenging malignancy because of its aggressive clinicopathological features. Although EC comprises several histologic subtypes, with esophageal squamous cell carcinoma (ESCC) being the most common, accounting for 96% of EC cases in Japan.^4^, ^5^ The rapid spread of ESCC explains in part why more than half of patients with ESCC have unresectable tumors or radiologically visible metastases at the time of diagnosis.^6^ Therefore, it is crucial to identify the mechanisms of acquisition of malignant traits in ESCC to help establish optimal treatment strategies.

Cancer stem cells (CSCs), which exhibit the principal properties of self‐renewal, clonal tumor initiation, and clonal long‐term repopulation potential, are present in EC as well as gastric and colorectal cancers.^7^, ^8^ CD44 is a transmembrane hyaluronan receptor and a major cell surface marker of CSCs.^9^, ^10^ Numerous CD44 isoforms (eg, CD44v3, CD44v6, and CD44v8‐10) are generated through alternative splicing.^11^ Interaction of CD44 variant 9 (CD44v9) with the glutamate‐cystine transporter increases intracellular levels of the antioxidant glutathione, which protects against reactive oxidant species (ROS). CD44v9‐induced glutathione is associated with resistance to chemotherapy and radiotherapy and increased tumor recurrence and metastasis.^12^, ^13^ To our knowledge, there is no previous report on the clinicopathological and prognostic significance of CD44v9 expression in patients with ESCC.

Epithelial‐mesenchymal transition (EMT), which involves conversion of epithelial cells to the mesenchymal phenotype,^14^ enhances the migration and invasiveness of tumor cells. EMT is mediated through signaling molecules and transcription factors such as transforming growth factor‐β (TGF‐β), hepatocyte growth factor (HGF), Zinc finger E‐box‐binding homeobox 1 (ZEB1), Snail, and Twist.^15^ We previously reported an association between EMT and poor prognosis of patients with ESCC;^16^, ^17^ however, the mechanism of induction of EMT and its effect on ESCCs is insufficiently characterized. Cancer cells that undergo EMT are frequently located at the tumor invasive front (TIF).^18^ For example, the transcription factor ZEB1 and the adhesion molecule E‐cadherin are expressed at increased and decreased levels, respectively, at the TIF.^19^ Therefore, investigating EMT at the TIF will help clarify its role in tumor cell invasiveness and metastatic traits.

Although the molecular mechanism remains unclear, there is an association between EMT and CSCs. For example, the expression of CSC markers such as CD44 is influenced by transcription factors associated with EMT,^20^, ^21^, ^22^ and induction of EMT leads to increased expression of stem cell markers by mammary epithelial cells.^22^ Furthermore, transcription factors associated with EMT regulate the acquisition of CSC properties and prevent tumor senescence.^22^, ^23^ We hypothesized that CD44v9 acts at the TIF through an association with EMT in ESCC. In this study, we aimed to clarify the relationships between CD44v9 expression, EMT, and clinicopathological features of patients with ESCC. In addition, using an ESCC cell line we showed that CD44v9 expression at the TIF was significantly associated with EMT and the malignant phenotype of ESCC.

## 2 MATERIALS AND METHODS

### 2.1 Patients

**Figure 1:**
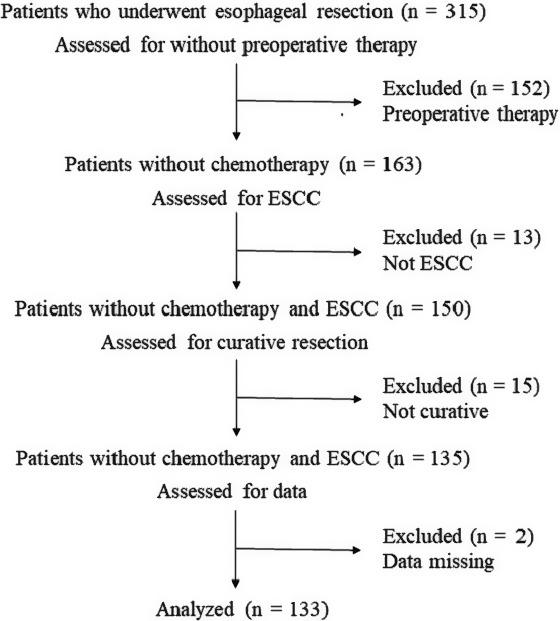
Flowchart depicting the patient selection process

Among 315 patients who underwent esophageal resection at the Department of Surgery and Science, Graduate School of Medical Sciences, Kyushu University between January 2002 and December 2012, 133 ESCC patients who underwent curative resection without preoperative therapies were included in the present study (Figure 1). In this study, we focused on CD44v9 expression at the TIF. We therefore excluded patients who underwent preoperative therapy because such therapies might affect intrinsic morphology at the TIF, resulting in inappropriate evaluation. The clinicopathological features of these patients included age at surgery, sex, smoking status, tumor differentiation, pathological depth of tumor invasion, pathological lymph node metastasis, and lymphovascular invasion. The research conforms to the provisions of the Declaration of Helsinki.

### 2.2 Immunohistochemistry

Immunohistochemistry (IHC) was performed using 4‐µm‐thick paraffin‐embedded tissue sections. Paraffin‐embedded blocks containing the site of the deepest tumor invasion in primary tumors (PTs) and all metastatic lymph nodes (mLNs) were chosen. The sections were deparaffinized in xylene and dehydrated in an ethanol series. For antigen retrieval, specimens in citrate buffer (pH 6.0) were heated in an autoclave at 105°C for 10 minutes. The sections were incubated for 30 minutes in 0.3% hydrogen peroxidase in methanol to deactivate endogenous peroxidases. After blocking the nonspecific binding of antibodies, the specimens were incubated with a rat anti‐CD44v9 monoclonal antibody (LKG‐M001, 1:5000 dilution; COSMO BIO CO LTD, Tokyo, Japan) and a rabbit anti‐E‐cadherin monoclonal antibody (24E10, 1:1000 dilution; Cell Signaling Technology Japan, Tokyo, Japan) at 4°C overnight. Immune complexes were detected using a DAKO EnVision Detection System (Dako, Glostrup, Denmark). The sections were reacted with 3,3′‐diaminobenzidine, counterstained with hematoxylin, and mounted.

### 2.3 Evaluation of IHC data

The samples were divided into a non‐EMT group and an EMT group. We defined the EMT group as tumor cells with decreased expression of E‐cadherin and morphological features of EMT at the TIF, as described previously.^16^ Decreased expression of E‐cadherin was defined as tumor cells at the TIF that exhibited undetectable or weak E‐cadherin staining compared with normal epithelial cells. We evaluated tumor budding at the TIF, which was defined as the presence of single isolated tumor cells or small clusters of cells in the stroma.^24^ EMT morphology was defined as five or more tumor buds at the TIF observed in high‐power fields (HPFs). This cut‐off value has been used to define positive budding in colon cancer and ESCC.^24^, ^25^, ^26^

Carcinoma cells that expressed membrane‐associated CD44v9 were defined as CD44v9‐positive, and the proportion of tumor cells that expressed CD44v9 was used to define CD44v9 scores as follows: 0 (absent), 1+ (<5%), 2+ (5%‐75%), and 3+ (>75%), as described previously.^27^ The intensity of CD44v9 expression was not used for evaluation. To compare CD44v9 expression at each tumor site, the proportion of CD44v9‐positive cells at the center or TIF of a PT was evaluated using the CD44v9 score of three HPFs. We designated a sample as CD44v9‐positive when the CD44v9 score was 3+ at the TIF, as previously described.^27^ To compare CD44v9 expression levels in PTs and mLNs, we used the CD44v9 score to evaluate the proportion of CD44v9‐positive cells in whole tumor sections of PTs and mLNs. When multiple mLNs were present, the LN with the highest CD44v9 expression level was used for evaluation.

### 2.4 Cell culture

Human ESCC cell lines (TE1, 3, 5, 6, 8, 11, 12, 13) were cultured in SILAC RPMI‐1640 (Thermo Fisher Scientific, Waltham, MA, USA), supplemented with 10% fetal bovine serum (FBS) (Biological Industries, Cromwell, CT, USA), at 37°C in an atmosphere containing 5% CO_2_.

A CD44v9‐specific siRNA^28^ was synthesized by Thermo Fisher Scientific. Among cell lines tested the expression of CD44v9 was highest in TE6 cells, which were therefore selected for further study. TE6 cells were seeded in a 6‐well plate (0.25 × 10^6^ cells per well) and reverse transfected with 30 nmol of CD44v9 siRNA in the presence of Lipofectamine RNAimax reagent (Thermo Fisher Scientific) for 72 hour, according to the procedure provided by the manufacturer. A duplex Stealth RNAi (siCONT) was used as a nontargeting siRNA.

To induce EMT, TE6 cells were seeded in a 6‐well plate (0.25 × 10^6^ cells per well) and treated with TGF‐β1 (final concentration of 20 ng/mL) (Invitrogen, Carlsbad, CA, USA) for 72 hour.

### 2.5 Western blot analysis

Proteins were extracted from cells using RIPA Buffer (Nacalai Tesque, Kyoto, Japan). The lysate was centrifuged at 15 000 *g* for 5 minutes at 4°C. Western blotting was performed using iBind Western Systems (Thermo Fisher Scientific) according to the procedure provided by the manufacturer. Immune complexes were visualized using an Amersham Imager600 (GE Healthcare, Little Chalfont, UK). The primary antibodies used were as follows: anti‐CD44v9 (LKG‐M001, 1:1000 dilution; COSMO BIO LTD), glyceraldehyde 3‐phosphate dehydrogenase (GAPDH) (GTX100118, 1:1000 dilution; GeneTex Inc, Irvine, CA, USA), anti‐E‐cadherin (24E10, 1:1000 dilution; Cell Signaling Technology Japan, Tokyo, Japan), and anti‐vimentin (D21H3, 1:1000 dilution; Cell Signaling Technology Japan).

### 2.6 Cell proliferation and intracellular ROS level assays

At 48 hour after siCD44v9 transfection, TGF‐β treatment, or combined siCD44v9 transfection and TGF‐β treatment, TE6 cells were seeded at 5000 cells/well in 96‐well plates and incubated at 37°C in an atmosphere containing 5% CO_2_. At the time of plating (0 hour) and 48 hour after incubation, cell growth rates and intracellular ROS levels were assessed using a CellTiter‐Glo Luminescent Cell Viability Assay kit (Promega, Madison, WI, USA) and ROS‐Glo H_2_O_2_ assay kit (Promega) according to the manufacturer's instructions. The 48‐hour cell growth rates were normalized and expressed as fold change in cell viability from 0 hour to 48 hour. The intracellular ROS levels were normalized to cell viability.

### 2.7 Cell migration and invasion assays

Cell migration and invasion assays were performed using Transwell insert chambers (Corning, New York, NY, USA) according to the manufacturer's instructions with minor modifications. TE6 cells (2.0 × 10^6^ per well) were transferred to serum‐free RPMI‐1640 in the upper chamber 48 hour after siRNA transfection, TGF‐β treatment, or combined treatment. RPMI‐1640 containing 20% FBS was added to the lower chamber as a chemoattractant. Following incubation for 48 hour at 37°C in an atmosphere containing 5% CO_2_, cells on the upper surface of the membrane were removed using a cotton swab, and migratory cells on the bottom of the membrane were stained using the Diff‐Quik protocol (Sysmex Corporation, Kobe, Japan). Migratory cells were observed using a light microscope. Cells were counted in three random fields (100× magnification), and the data are expressed as the average number of cells per field of view. All assays were performed three times. The invasion assay employed identical methods, except that the cells were placed in the upper chamber containing a Matrigel‐coated membrane (BD Biosciences, Franklin Lakes, NJ, USA).

### 2.8 Statistical analysis

JMP software (ver. 13.0, SAS Institute Inc, Cary, NC, USA) was used to perform statistical analyses. The associations between CD44v9 expression and clinicopathological factors were analyzed using the chi‐square test. Overall survival (OS) was defined from the day of surgery to the day of death or the most recent follow‐up visit, and recurrence‐free survival (RFS) was defined from the day of surgery to the day of death, tumor recurrence, or the most recent follow‐up visit. Kaplan‐Meier curves and univariate analysis were used to estimate the distributions of OS and RFS, and statistical significance was determined using the log‐rank test. After univariate analysis of the factors affecting OS and RFS, only significant variables (*P* < 0.05) were incorporated in multivariate analysis using the Cox proportional hazard model. CD44v9 expression in the PT versus mLNs was compared using the paired *t* test. A value of *P* < 0.05 was considered significant.

## 3 RESULTS

### 3.1 CD44v9 expression in ESCC

**Figure 2:**
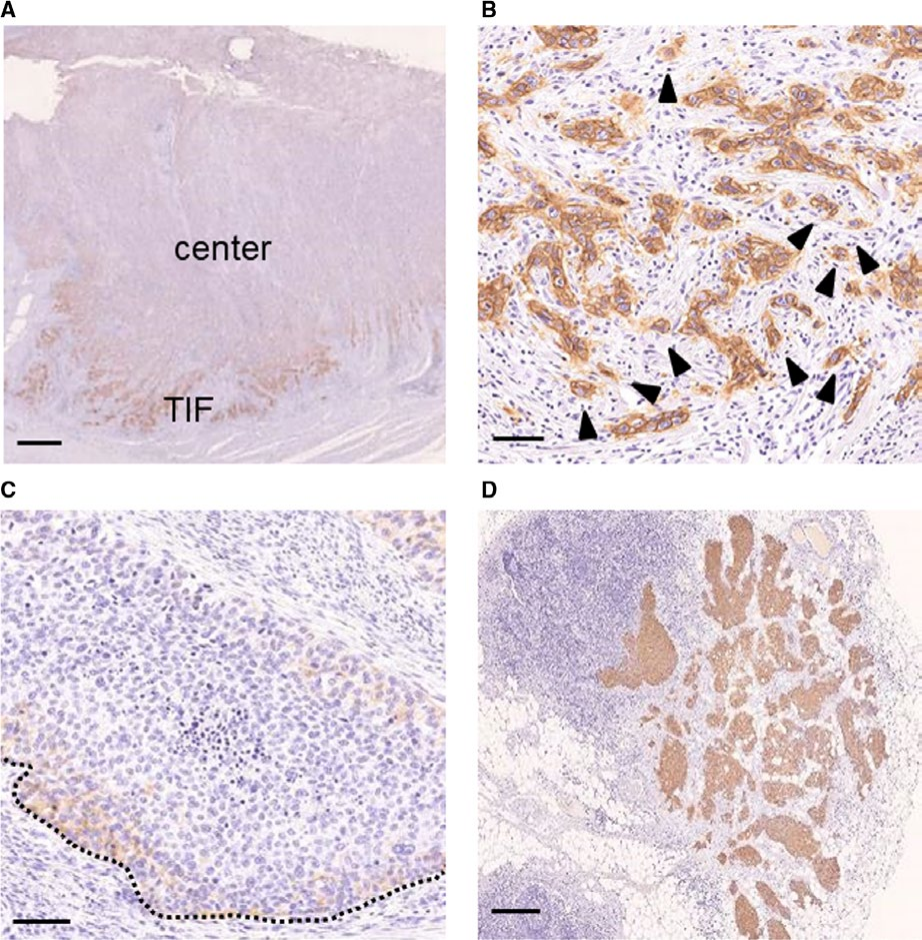
Representative immunohistochemical analyses of CD44v9. (A) CD44v9 expression in whole tumor section. CD44v9 expression was higher at the TIF compared with the center of the tumor. (B) Strong CD44v9 expression at the TIF in the EMT group. (C) CD44v9 expression in mLNs in the same patient as panel (A). (D) Weak CD44v9 expression at the TIF in the non‐EMT group. CD44v9 expression was higher in the EMT group compared with the non‐EMT group and higher at the TIF and mLNs compared with the whole primary tumor section. The dotted line indicates the TIF and black arrows indicate budding tumor cells. Scale bars: (A) 1 mm, (B, C) 100 µm, and (D) 500 µm. EMT, epithelial‐mesenchymal transition; mLN, metastatic lymph node; TIF, tumor invasive front

Based on morphological features at the TIF (Figure S1A and B) and the expression of E‐cadherin (Figure S1C‐E), 40 patients (30%) were assigned to the EMT group. We used the CD44v9 score to evaluate differences in CD44v9 expression according to the site within the tumor (Figure 2A). Figure 2B and C show representative images of CD44v9 expression at the TIF of the EMT group and non‐EMT group, respectively. As shown in Table [Link], [Link], 19 (14%) and 59 (44%) samples were scored 3+ at the center of the tumor and the TIF, respectively (*P* < 0.001). CD44v9 expression at the TIF was significantly higher in the EMT group compared with the non‐EMT group (*P* < 0.001) (Table S2).

### 3.2 Difference in CD44v9 expression between the PT and mLNs

Figure 2A and D show representative images of CD44v9 expression of the PT and mLNs of one patient. Among 133 patients, mLNs were observed in 60 (45%) cases (Table S3), and the CD44v9 score was significantly higher in mLNs compared with the PTs in these 60 patients (*P* = 0.0017).

### 3.3 Clinicopathological features of patients

Table 1 shows the association between CD44v9 expression at the TIF and the characteristics of patients included in this study. Among 133 patients, 59 (44%) were defined as CD44v9‐positive. CD44v9 expression at the TIF was associated with deeper tumor invasion (*P* = 0.029) and EMT (*P* < 0.001). Other factors were not significantly associated.

**Table 1:** Association between CD44v9 expression at the TIF and clinicopathological features of patients with surgically resected ESCC

Factors	CD44v9		*P* value
	Negative n = 74 (%)	Positive n = 59 (%)	
Age (y)			
<70	54 (73)	46 (78)	0.51
≥70	20 (27)	13 (22)	
Sex			
Female	7 (9)	6 (10)	0.89
Male	67 (91)	53 (90)	
Smoking status			
Never‐smoker	23 (31)	14 (24)	0.14
Smoker	51 (69)	43 (73)	
Unknown	0 (0)	2 (3)	
Differentiation of SCC			
Well/moderately	58 (78)	52 (88)	0.13
Poorly	16 (22)	7 (12)	
Pathological depth of tumor invasion			
<T3	49 (66)	28 (47)	0.029
≥T3	25 (34)	31 (53)	
Pathological lymph node metastasis			
Negative	43 (58)	30 (51)	0.40
Positive	31 (42)	29 (49)	
Lymphatic invasion			
Negative	44 (59)	31 (53)	0.42
Positive	30 (41)	28 (47)	
Vascular invasion			
Negative	56 (76)	40 (68)	0.31
Positive	18 (24)	19 (32)	
EMT			
Negative	67 (91)	26 (44)	<0.001
Positive	7 (9)	33 (56)	

TIF, tumor invasive front; ESCC, esophageal squamous cell carcinoma; EMT, epithelial‐mesenchymal transition.

### 3.4 CD44v9 expression at the TIF and patient outcomes

**Figure 3:**
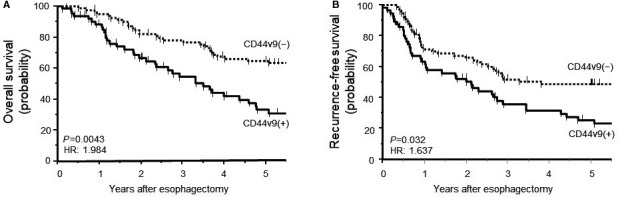
Kaplan‐Meier analysis of survival as a function of CD44v9 expression. (A) Overall survival and (B) recurrence‐free survival of patients were significantly shorter for patients with CD44v9 expression compared with those without (*P* = 0.0043 and *P* = 0.032, respectively). HR, hazard ratio

To evaluate the prognostic significance of CD44v9 expression at the TIF, we examined the association between CD44v9 expression at the TIF and survival. CD44v9‐positive patients experienced significantly lower 5 year OS (hazard ratio [HR] 1.984; *P* = 0.0043) and RFS (HR 1.637; *P* = 0.032) rates compared with those without CD44v9 expression (Figure 3). Multivariate analyses revealed that lymphatic invasion and CD44v9 expression at the TIF were independent prognostic factors of OS (*P* = 0.0068 and *P* = 0.0079, respectively) (Table 2). The depth of tumor invasion, lymphatic invasion, and CD44v9 expression at the TIF were independent prognostic factors of RFS (*P* = 0.0024, *P* < 0.001, and *P* = 0.0212, respectively) (Table 2).

**Table 2:** Univariate and multivariate analyses of RFS and OS in patients with ESCC who underwent esophagectomy

Factors		OS				RFS			
		Univariate analysis		Multivariate analysis		Univariate analysis		Multivariate analysis	
		HR (95%CI)	*P* value	HR (95%CI)	*P* value	HR (95%CI)	*P* value	HR (95%CI)	*P* value
Age (y)	≧70/<70	1.449 (0.840‐2.410)	0.176			1.326 (0.774‐2.182)	0.294		
Sex	Female/male	0.829 (0.320‐1.764)	0.652			0.680 (0.263‐1.440)	0.339		
Differentiation of ESCC	Poor/well moderate	1.169 (0.624‐2.048)	0.608			1.284 (0.714‐2.180)	0.388		
Pathological depth of tumor invasion	T3‐4/T0‐2	2.873 (1.776‐4.701)	<0.001	1.654 (0.952‐2.900)	0.0740	3.694 (2.322‐5.947)	<0.001	2.278 (1.338‐3.904)	0.0024
Pathological lymph node metastasis	Positive/negative	2.869 (1.766‐4.715)	<0.001	1.501 (0.832‐2.725)	0.178	3.181 (2.009‐5.099)	<0.001	1.373 (0.779‐2.425)	0.273
Lymphatic invasion	Positive/negative	2.872 (1.783‐4.691)	<0.001	2.128 (1.231‐3.719)	0.0068	3.913 (2.441‐6.360)	<0.001	3.071 (1.778‐5.354)	<0.001
Vascular invasion	Positive/negative	2.175 (1.315‐3.532)	0.0029	1.233 (0.708‐2.123)	0.455	2.649 (1.647‐4.201)	<0.001	1.250 (0.729‐2.127)	0.414
EMT	Positive/negative	1.479 (0.879‐2.421)	0.137			1.392 (0.848‐2.227)	0.186		
CD44v9 at TIF	Positive/negative	1.984 (1.227‐3.217)	0.0054	1.954 (1.193‐3.208)	0.0079	1.637 (1.034‐2.582)	0.0345	1.751 (1.088‐2.817)	0.0212

RFS, recurrence‐free survival; OS, overall survival; ESCC, esophageal squamous cell carcinoma; HR, hazard ratio; CI, confidence interval EMT, epithelial‐mesenchymal transition; TIF, tumor invasive front.

### 3.5 Causal relationship between CD44v9 expression and EMT

**Figure 4:**
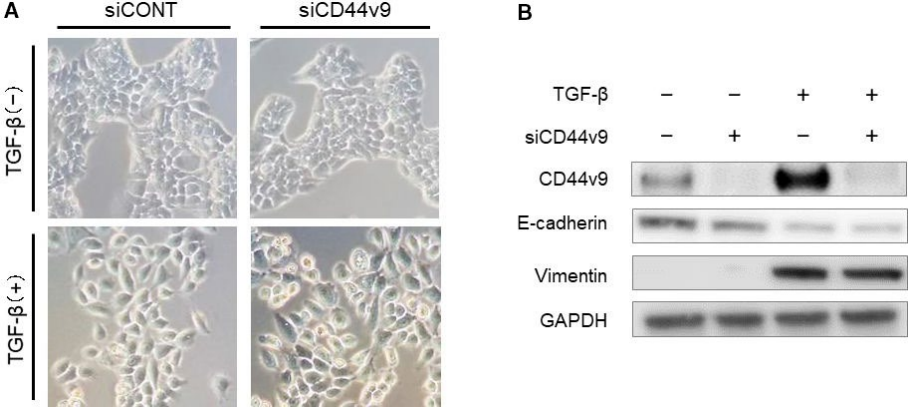
CD44v9 expression in ESCC cells induced to undergo EMT. (A) Morphological features of TE6 cells. (B) Western blot analysis of CD44v9, E‐cadherin, and vimentin in TE6 cells. TGF‐β converted TE6 cells to a spindle‐like mesenchymal morphology. The expression of E‐cadherin was decreased, and the expression of vimentin and CD44v9 was increased, by TGF‐β stimulation. In contrast, cell morphology and the expression of CD44v9, E‐cadherin, and vimentin were not changed in cells transfected with siCD44v9. ESCC, esophageal squamous cell carcinoma; EMT, epithelial‐mesenchymal transition; GAPDH, glyceraldehyde 3‐phosphate dehydrogenase; TGF‐β, transforming growth factor‐β

We used an ESCC cell line to assess the causal relationship between CD44v9 expression and EMT. After treatment of TE6 cells with TGF‐β to induce EMT, the cobblestone‐like epithelial morphology was converted to a spindle‐like mesenchymal morphology. In contrast, siCD44v9‐transfected cells retained a cobblestone‐like morphology. In addition, TE6 cells treated with TGF‐β and siCD44v9 retained a spindle‐like mesenchymal morphology similar to that of cells treated with TGF‐β alone, suggesting that siCD44v9 did not affect morphology (Figure 4A). Although the expression of CD44v9 and vimentin increased, and expression of E‐cadherin decreased, in cells treated with TGF‐β, the expression of E‐cadherin and vimentin was unchanged in siCD44v9‐transfected cells. CD44v9 expression was undetectable in TE6 cells treated with TGF‐β and siCD44v9 (Figure 4B). In contrast, the expression of E‐cadherin and vimentin was not affected by siCD44v9 in cells treated with TGF‐β (Figure 4B). These results suggest that CD44v9 expression was induced by EMT.

### 3.6 The role of CD44v9 in tumor cell proliferation and intracellular ROS level

To evaluate the contribution of CD44v9 to cell proliferation and intracellular ROS level, we treated TE6 cells with siCD44v9, TGF‐β, or combined siCD44v9 and TGF‐β. The 48‐h cell growth rates were not changed after treatment with siCD44v9, TGF‐β, or both. The intracellular ROS level was increased 96 hours after treatment with siCD44v9 or both siCD44v9 and TGF‐β (*P* = 0.0051 and 0.0051, respectively). In contrast, the intracellular ROS level was not affected by treatment with TGF‐β alone (Figure S2).

### 3.7 The role of CD44v9 in tumor cell migration and invasion

**Figure 5:**
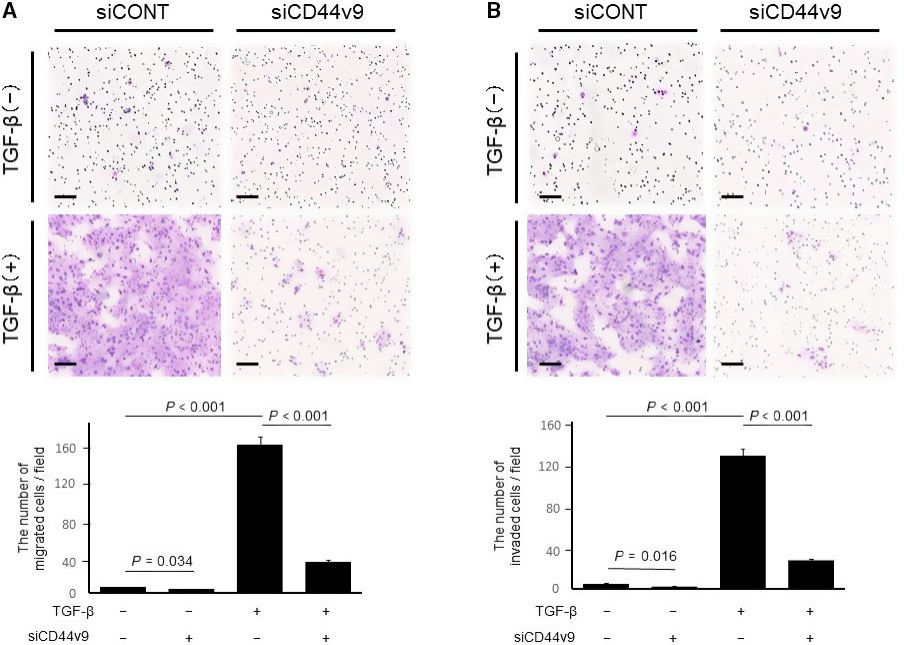
Migration and invasiveness of ESCC cells transfected with siCD44v9. Representative (A) migration and (B) invasion assays. Migration and invasiveness of TE6 cells were enhanced by TGF‐β treatment and inhibited in siCD44v9‐transfected cells. Each experiment was independently repeated three times. Data are presented as the mean ± standard deviation. ESCC, esophageal squamous cell carcinoma; siCONT, stealth RNAi; TGF‐β, transforming growth factor‐β

We hypothesized that CD44v9 expression promotes tumor cell migration and invasion because CD44v9 expression was higher at the TIF and its expression at the TIF was associated with the depth of tumor invasion (Table [Link], [Link] and Table 1). To evaluate the contribution of CD44v9 to cell migration and invasion, we treated TE6 cells with siCD44v9, TGF‐β, or combined siCD44v9 and TGF‐β. The number of migrating and invading cells was significantly decreased in TE6 cells treated with siCD44v9 compared with cells transfected with siCONT (*P* = 0.034 and *P* = 0.016, respectively) and was significantly increased by treatment with TGF‐β (both *P* < 0.001). In contrast, the number of migrating and invading cells was significantly decreased for cells treated with siCD44v9 and TGF‐β compared with TGF‐β treatment alone (both *P* < 0.001) (Figure 5). These results indicate that CD44v9 enhanced tumor migration and invasion through a mechanism independent of EMT.

## 4 DISCUSSION

In this study, we analyzed CD44v9 expression at the tumor invasive front in patients with ESCC who were not administered preoperative therapy and investigated the association between CD44v9 expression and patients’ clinicopathological features and EMT. Immunohistochemical analysis demonstrated that CD44v9 expression was significantly higher at the TIF and was significantly associated with the depth of tumor invasion, thus serving as an independent prognostic factor. We further demonstrated that expression of CD44v9 in an ESCC cell line was increased by TGF‐β‐induced EMT. Furthermore, siCD44v9‐transfected ESCC cells showed decreased migration and invasiveness.

To our knowledge, this is the first study to report the significance of CD44v9 expression in ESCC. CD44v9 expression is significantly associated with poor prognosis of patients with gastric, bladder, and liver cancers, as well as other cancers.^28^, ^29^, ^30^, ^31^ Although most studies of CD44v9 examined its expression according to the intensity of staining or the proportion of CD44v9‐positive cells in whole tumor sections, several studies have reported levels of CD44v9 expression at the TIF.^28^, ^29^ For example, in non‐muscle invasive bladder cancer expression of CD44v9 is localized to the TIF.^28^ In gastric cancer, expression of CD44v6 and CD44v9 at the TIF is more strongly associated with poor prognosis than expression in whole tumor sections.^29^

In the present study, CD44v9 expression in whole tumor sections was not associated with patient outcomes and clinicopathological features (data not shown). In contrast, CD44v9 expression at the TIF was significantly associated with the depth of tumor invasion and poor OS and RFS (Table 1 and Figure 3). These results suggested that expression of CD44v9 at the TIF, but not in whole tumor sections, may play an essential role in the acquisition of clinicopathological malignant traits. The significance of CD44v9 expression at the TIF may differ according to the type of cancer. The association between EMT and poor prognosis in ESCC is currently unclear. In this study, CD44v9 expression at the TIF was associated with EMT and poor prognosis (Table 1 and Figure 3) whereas EMT was not associated with survival (Table 2). However, CD44v9 expression increased following EMT activation (Figure 4). It is reported that elevated CD44v9 expression increases antioxidant capacity and is associated with tumor invasion and metastasis.^12^, ^32^ Therefore, CD44v9 expression at the TIF in addition to EMT may accelerate tumor malignant traits.

In this study, we defined EMTbased on decreased expression of E‐cadherin and morphological features of the TIF. Tumor budding is associated with a type of diffusely infiltrative growth observed in many gastrointestinal cancers and is recognized as a measure of cancer aggressiveness.^33^ Furthermore, evidence indicates that tumor budding is representative of EMT^34^; for example, studies of transcriptional differences between the center of a tumor and tumor buds in oral squamous cell carcinoma and colorectal cancer^35^, ^36^ demonstrate that cells in the tumor buds exhibit a gene expression signature more characteristic of EMT phenotype compared with cells in the center of the tumor. Moreover, tumor budding displaying a spindle‐like morphology is associated with decreased expression of E‐cadherin and increased expression of ZEB1.^19^ In ESCC, the EMT status, defined as a high vimentin to E‐cadherin RNA expression ratio in cancerous tissues, is associated with tumor budding.^37^ Further studies are required to establish the biological and clinical significance of tumor budding in this highly aggressive disease.

The identity of the signaling pathway that links the EMT with the acquisition of CSC properties is unknown, although an association between these phenotypes is supported by compelling evidence. Cancer cells at the tumor invasive front are frequently exposed to several cytokines (eg, TGF‐β, HGF) and enzymes (eg, matrix metalloproteinase).^38^, ^39^, ^40^ An activated EMT program establishes autocrine signaling loops, including those of the TGF‐β and Wnt‐β‐catenin signal transduction pathways, which contribute to the CSC phenotype.^41^ EMT program also contributes to CSC function through several intracellular signaling pathways. For example, Twist, which is an inducer of EMT, prevents tumor senescence,^23^ and overexpression of Twist enhances in vitro tumor‐sphere formation and in vitro tumor seeding.^22^ Similarly, knockdown of Snail, which also induces EMT, reduces tumor‐sphere formation in head and neck carcinoma.^42^ As mentioned above, some reports have revealed that several cytokines and transcriptional factors that induce EMT also contribute to induction of a CSC phenotype. In the present study, IHC analysis detected high levels of CD44v9 expression in the TIF, particularly in tumor buds (Tables S1 and S2), and TGF‐β stimulation induced high expression of CD44v9 in ESCC cells (Figure 4B). Several studies have reported a positive role of TGF‐β in the CSC population by promoting or sustaining stemness.^43^, ^44^, ^45^ Although it is not clear how expression of the CSC markers CD44 or CD44v9 is regulated, activation of the EMT program by TGF‐β at the TIF may contribute to tumor stemness, and this tumor microenvironment may increase the expression of CD44v9 expression at the TIF.

During invasion and metastasis, cancer cells are exposed to environmental stressors such as ROS.^13^ Therefore, inhibition of such oxidative stress at potential sites of invasion or metastasis may be required for cancer cells to establish invasive or metastatic lesions. The interaction of CD44v8‐10 with the cystine transporter stabilizes intracellular GSH levels, potentiating the ability of cancer cells to defend themselves against ROS.^12^ CD44v9 expression was significantly higher at the TIF compared with the center of the tumor (Table 1), suggesting that higher CD44v9 expression at the TIF may reflect an increased ability of CD44v9‐positive tumor cells to invade the stroma. Intracellular GSH production, regulation, and utilization are regulated by several antioxidants and enzymes.^46^, ^47^, ^48^, ^49^, ^50^ In addition, the effect of ROS on tumor cell fate depends on the ROS level;^46^, ^51^ low levels of ROS provide a beneficial effect, supporting cell proliferation, and survival pathways, whereas high levels of ROS cause detrimental oxidative stress that can lead to cell death.^46^ For these reasons, we assessed intracellular ROS levels to investigate whether CD44v9 inhibition directly affects ROS production. We found that the intracellular ROS level was increased 96 hours after siCD44v9 transfection and after combined siCD44v9 transfection and TGF‐β treatment (*P* = 0.0051 and 0.0051, respectively). In contrast, the intracellular ROS level was not affected by TGF‐β treatment alone (Figure S2).

These findings are supported by our demonstration that inhibition of CD44v9 expression by siCD44v9 decreased tumor cell migration and invasiveness (Figure 5). The siCD44v9‐induced suppression of resistance to ROS, which tumor cells are exposed to during invasion, may inhibit the migration and invasiveness of tumor cells. Thus, CD44v9 may represent a therapeutic target for patients with ESCC. Sulfasalazine (SSZ), which has long been used to treat patients with rheumatoid arthritis and ulcerative colitis, specifically inhibits the cystine transporter.^52^ Studies to evaluate the effects of SSZ on patients with advanced gastric cancer and non‐squamous non‐small cell lung cancer have been conducted in Japan.^53^, ^54^, ^55^ The development of anti‐CD44v9 targeting therapy may therefore represent a candidate novel treatment strategy for managing patients with ESCC.

The present study has some limitations. First, we defined the non‐EMT and EMT groups based only on E‐cadherin expression and morphological features at the TIF. However, this definition of EMT has not been established in tissue specimens and an appropriate definition of EMT at the TIF in resected specimens should be established. Second, this was a retrospective study of patients treated at a single institution. However, we are unaware of any other studies that focus on CD44v9 expression in patients with ESCC and therefore our findings provide useful novel information that suggests the value of further studies on the role of CD44v9 in ESCC. Third, we revealed that CD44v9 inhibition decreased tumor cell invasion capacity in vitro and high CD44v9 expression was associated with deeper tumor invasion in human material; however, data on tumor invasion in an animal model are lacking and further examinations in animal models are required.

In conclusion, CD44v9 expression at the TIF was associated with EMT, tumor invasion, and the malignant potential of ESCC cells. We propose that acquisition of CSC properties induced by EMT at the TIF contributes to the migration and invasiveness of cancer cells. Together, these findings strongly suggest that CD44v9 represents a novel prognostic biomarker and a potential therapeutic target for ESCC.

## CONFLICT OF INTEREST

None Declared.
