# Supplementary figures and images for: CD44v9 is associated with epithelial‐mesenchymal transition and poor outcomes in esophageal squamous cell carcinoma
Source: Cancer Med. 2018 Nov 26;7(12):6258–68. doi: 10.1002/cam4.1874 (PMC6308082; doi:10.1002/cam4.1874)

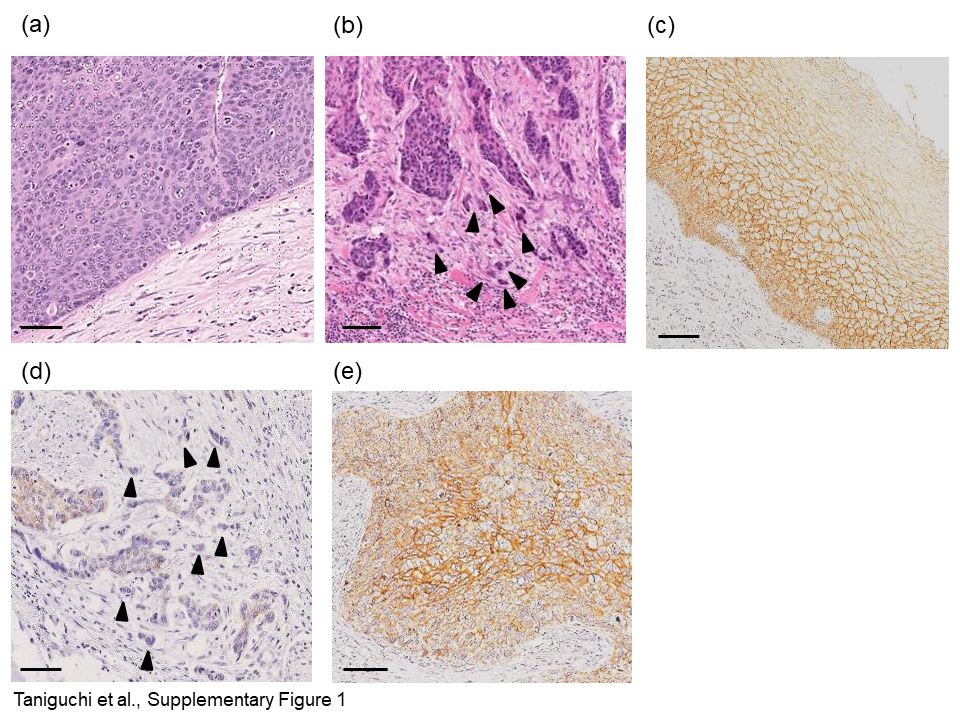

Supplement: Supplementary file 1 [file CAM4-7-6258-s001.tif]

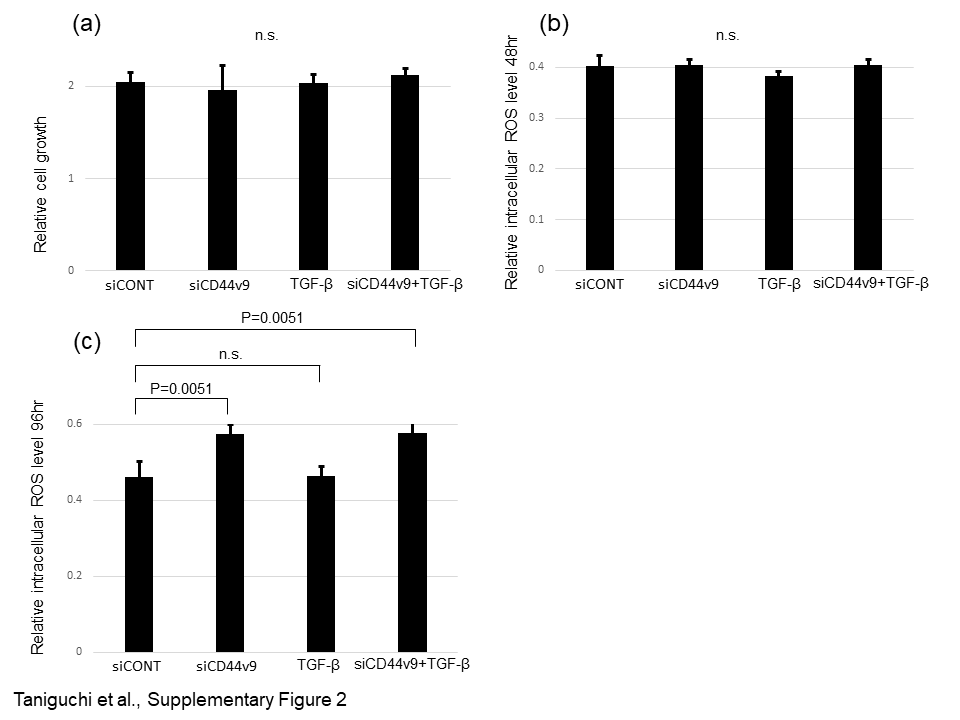

Supplement: Supplementary file 2 [file CAM4-7-6258-s002.tif]
