# Supplementary material for: CD44v9 is associated with epithelial‐mesenchymal transition and poor outcomes in esophageal squamous cell carcinoma
Source: Cancer Med. 2018 Nov 26;7(12):6258–68. doi: 10.1002/cam4.1874 (PMC6308082; doi:10.1002/cam4.1874)
Supplement: Supplementary file 3 [file CAM4-7-6258-s003.docx]

**Table S1**. CD44v9 expression at the center of the tumor and the tumor invasive front in ESCC.

| CD44v9 score | Center of tumor  n=133 (%) | TIF  n=133 (%) | *P* value |
| --- | --- | --- | --- |
| 0 | 35 (26) | 14 (11) | <0.001 |
|  |  |  |  |
| 1+ | 42 (32) | 27 (20) |  |
|  |  |  |  |
| 2+ | 37 (28) | 33 (25) |  |
|  |  |  |  |
| 3+ | 19 (14) | 59 (44) |  |

TIF, tumor invasive front; ESCC, esophageal squamous cell carcinoma

**Table S2**. CD44v9 expression at the TIF in the non-EMT and EMT groups.

| CD44v9 score | non-EMT  n=93 (%) | EMT  n=40 (%) | *P* value |
| --- | --- | --- | --- |
| 0 | 14 (15) | 0 (0) | <0.001 |
|  |  |  |  |
| 1+ | 26 (28) | 1 (3) |  |
|  |  |  |  |
| 2+ | 27 (29) | 6 (15) |  |
|  |  |  |  |
| 3+ | 26 (28) | 33 (83) |  |

TIF, tumor invasive front; EMT, epithelial mesenchymal transition; ESCC, esophageal squamous cell carcinoma

**Table S3**. CD44v9 expression in resected PTs and mLNs in patients with mLNs.

| CD44v9 score | PT versus LN | | |
| --- | --- | --- | --- |
|  | PT  n=60 (%) | LN  n=60 (%) | *P* value |
| 0 | 2 (3) | 1 (2) | 0.0017 |
|  |  |  |  |
| 1+ | 18 (30) | 10 (17) |  |
|  |  |  |  |
| 2+ | 23 (38) | 18 (30) |  |
|  |  |  |  |
| 3+ | 17 (28) | 31 (52) |  |

PT, primary tumors; mLN, metastatic lymph node
